# *Porphyromonas gingivalis* and Alzheimer disease: Recent findings and potential therapies

**DOI:** 10.1002/JPER.20-0104

**Published:** 2020-08-06

**Authors:** Mark I. Ryder

**Affiliations:** ^1^ Division of Periodontology Department of Orofacial Sciences School of Dentistry University of California San Francisco CA

**Keywords:** Alzheimer disease, COR388, gingipain, neuroinflammation, periodontitis, *Porphyromonas gingivalis*

## Abstract

Epidemiological studies have identified an association between periodontitis and Alzheimer disease (AD); however, the nature of this association has been unclear. Recent work suggests that brain colonization by the periodontal pathogen *Porphyromonas gingivalis* may link these two inflammatory and degenerative conditions. Evidence of *P. gingivalis* infiltration has been detected in autopsy specimens from the brains of people with AD and in cerebrospinal fluid of individuals diagnosed with AD. Gingipains, a class of *P. gingivalis* proteases, are found in association with neurons, tau tangles, and beta‐amyloid in specimens from the brains of individuals with AD. The brains of mice orally infected with P. gingivalis show evidence of *P. gingivalis* infiltration, along with various neuropathological hallmarks of AD. Oral administration of gingipain inhibitors to mice with established brain infections decreases the abundance of *P. gingivalis* DNA in brain and mitigates the neurotoxic effects of *P. gingivalis* infection. Thus, gingipain inhibition could provide a potential approach to the treatment of both periodontitis and AD.

## 1 INTRODUCTION

Over the past 15 years a possible association between Alzheimer disease (AD) and periodontitis has emerged. AD is a progressive neuroinflammatory and neurodegenerative disease of the brain,^1^ defined by the accumulation and deposition of beta‐amyloid, which has recently been identified as an antimicrobial peptide,^2^, ^3^ and the presence of neurofibrillary tangles composed of aggregates of aberrantly phosphorylated tau proteins. Periodontitis is an inflammatory condition involving oral dysbiosis and progressive destruction of tissues supporting the teeth and is associated with various systemic disorders.^4^, ^5^ However, establishing a definitive causal link between periodontitis and extraoral disease such as AD, versus a simple association between the two conditions, depends on satisfying three criteria.^6^ The first criterion is association, which depends on demonstrating that periodontitis occurs in conjunction with the second condition. The second depends on establishing a plausible biological mechanism whereby periodontitis could initiate or promote the associated condition. Such biological mechanisms that could strengthen this causal link would include studies on the translocation of bacteria from dental plaque into the bloodstream, resulting in their direct effects on other organ systems, as well as local and systemic changes in the host response to these bacteria. Third, and most difficult to satisfy, is to demonstrate that the treatment of periodontitis per se, or targeted therapies against specific periodontal microbial pathogens, in some way modifies or ameliorates the associated condition. Satisfying this third criterion requires well‐designed and adequately powered randomized controlled clinical trials to compare the effects of targeted therapies with matched placebo controls. Recent studies suggest that the periodontal pathogen *Porphyromonas gingivalis* may provide the mechanistic link between these two inflammatory and degenerative conditions.^7^

## 2 PERIODONTITIS AND AD: ASSOCIATION STUDIES

Several studies have demonstrated an association between AD and periodontitis. Some are straightforward epidemiological studies;^8^, ^9^, ^10^, ^11^, ^12^, ^13^, ^14^, ^15^ others are studies that link periodontitis with serum levels of beta‐amyloid^16^ or brain imaging for amyloid.^17^

Association studies attempting to establish a causal relationship between two conditions, however, suffer from what one might call the “chicken and egg problem”: which came first? And what might be the role of other possible mediating factors such as genetics, age, tobacco use? This is a real concern in attempting to untangle the relationship between periodontitis and AD.^18^ One possibility is that periodontitis precipitates or exacerbates AD; alternatively, individuals with AD may not practice optimal oral hygiene and, as a result, may accumulate more oral plaque and develop more severe periodontitis. A third possibility is that periodontitis and AD may result from the same common factor, such as an infectious agent, genetic susceptibility, lifestyle, etc.

Therefore, to conclude that a direct causal relationship between periodontitis and AD exists, it is necessary to address the criterion of identifying a plausible biological mechanism and that of demonstrating that treating periodontitis, or a periodontitis related pathogen, affects the development or progression of AD.

## 3 PERIODONTITIS AND AD: MECHANISTIC STUDIES

**FIGURE 1:**
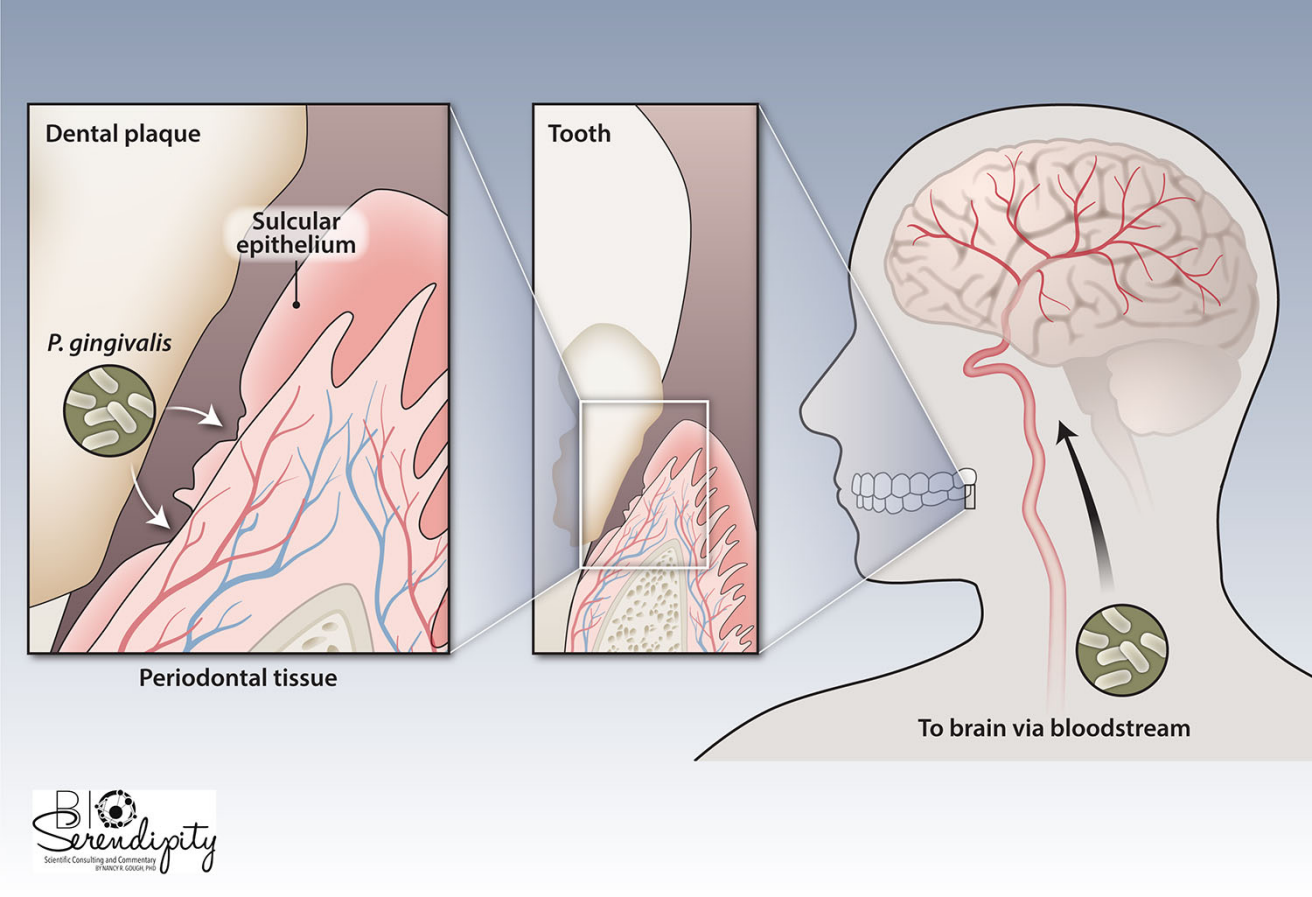
*P. gingivalis* can invade the periodontal tissues through the sulcular epithelium, gaining entry into the periodontal microcirculation where it can then spread through the bloodstream and colonize the brain. [Credit: Heather McDonald, BioSerendipity, LLC, Elkridge, MD]

*P. gingivalis* is a keystone pathogen in chronic periodontitis.^19^, ^20^ That is to say, it modulates the size and composition of the local microbial community to promote periodontitis and an inflammatory milieu. Moreover, it is capable of escaping into the bloodstream and colonizing extraoral tissues^21^, ^22^, ^23^, ^24^ (Figure 1). The gingipains are proteolytic enzymes essential for *P. gingivalis* survival.^25^, ^26^ Inhibiting gingipains may prevent *P. gingivalis* from thriving and/or proliferating.

Recent studies have identified *P. gingivalis* lipopolysaccharide^27^ and DNA^28^ in autopsy specimens from the brains of people who had AD. Moreover, *P. gingivalis* DNA was present in the cerebrospinal fluid and saliva of individuals with mild‐to‐moderate cognitive impairment, clinically diagnosed as having probable AD.^28^

**FIGURE 2:**
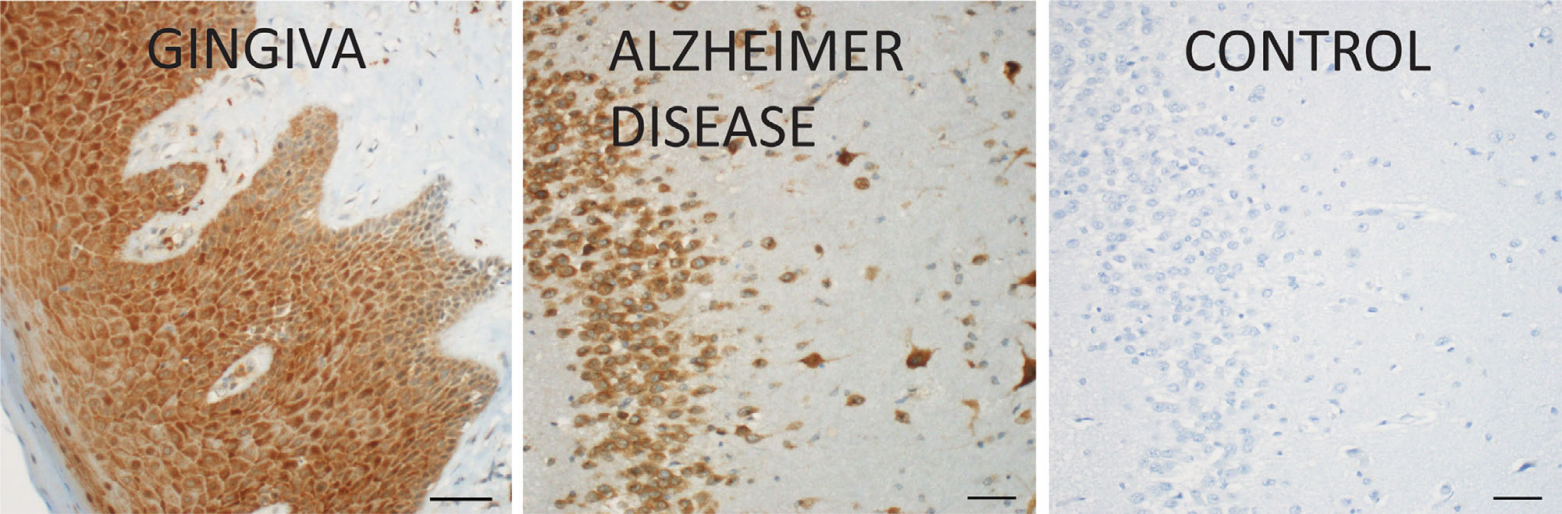
*P. gingivalis* gingipains localized in inflamed gingiva and the hippocampus region of an AD brain. Scale bar = 50 μm. Reprinted from Dominy et al.,^28^ which was published (and can be reproduced) under the terms of Creative Commons Attribution 4.0 License

Furthermore, Dominy et al. found that gingipains, which can be detected immunohistochemically in gingival tissue from individuals with periodontal disease,^28^ were present not only in gingiva, but in autopsy specimens of brain (Figure 2). Gingipains were more abundant in autopsy specimens of AD brain compared with specimens from control brain (from people who had not shown cognitive dysfunction) and their abundance correlated with that of tau and ubiquitin pathology.

Although the gingipains were localized throughout the brain, they were most prominent in regions associated with memory, such as the hippocampus. Moreover, immunofluorescence analyses revealed that gingipains colocalized with neurons, and were found in association with tau tangles and intracellular amyloid beta.

Studies in both wild‐type and apolipoprotein E (ApoE)‐/‐ mice support a role for *P. gingivalis* in explaining the possible link between periodontitis and AD.^28^, ^29^, ^30^, ^31^ The brains of mice orally infected with *P. gingivalis* show evidence of *P. gingivalis* infiltration, as well as neuroinflammation, amyloid plaques, activated microglia, tau tangles, and neurodegeneration. Gingipains can be detected in the brains of the infected mice, where they are localized in neurons, microglia, and astrocytes and are also found extracellularly.^28^, ^30^

## 4 PERIODONTITIS AND AD: THERAPEUTIC STUDIES

Mouse models of periodontitis‐AD also provide a system for testing an intriguing approach toward addressing the third criterion for assessing a link between the two conditions, that of showing that treating a periodontitis‐associated pathogen affects the development or progression of AD. Because the gingipains are essential for *P. gingivalis* survival, their inhibition could provide a mechanism for treating periodontitis; if *P. gingivalis* provides a link between periodontitis and AD, gingipain inhibition should affect AD pathology as well.

Dominy et al.^28^ developed a series of small molecule gingipain inhibitors and determined their effects on gingipain neurotoxicity in the brains of wild‐type mice and in an in vitro system using a neuronally‐differentiated neuroblastoma cell line. Pretreatment of mice with gingipain inhibitors protected their hippocampal neurons from the neurotoxic effects of injecting gingipain directly into the hippocampus. Furthermore, gingipain inhibitors protected the cultured cells from the toxic effects of *P. gingivalis*, whereas antibiotics, such as moxifloxacin and doxycycline, or semagacestat, a drug that inhibits the production of beta‐amyloid, did not.

Notably, oral administration of a gingipain inhibitor to mice in which brain infection by *P. gingivalis* had already been established decreased the abundance of *P. gingivalis* DNA in brain, as well as that of beta‐amyloid and the inflammatory mediator tumor necrosis factor‐α. Moreover, administration of gingipain inhibitors mitigated the neurotoxic effects of *P. gingivalis* infection, so that significantly more hippocampal neurons could be detected in the brains of infected mice treated with the inhibitor than in the brains of untreated infected mice.

These data suggest that gingipain inhibitors may provide a promising approach to the treatment of both periodontitis and AD. More definitive evidence will depend on the results of clinical trials assessing the effects of the gingipain inhibitors on periodontitis and AD. Several Phase 1a/b Food and Drug Administration (FDA) clinical trials of one of these compounds, COR388, have been completed (https://clinicaltrials.gov/ct2/show/NCT03418688).^32^ These Phase 1 a/b trials demonstrated that administration of COR388 for 28 days was well tolerated, rapidly absorbed to reach the desired therapeutic concentrations, and reduced the concentration of ApoE fragments in the cerebrospinal fluid, a marker of AD.^33^ A Phase 2/3 study (https://clinicaltrials.gov/ct2/show/NCT03823404)^34^ is now underway.

## DISCLOSURES

COR388 and the other gingipain inhibitors discussed in this article were developed at Cortexyme, Inc., South San Francisco, CA. Dr. Ryder is on the Clinical Advisory Board of Cortexyme, Inc. and is a paid consultant for them.
